# Proposal of a Vaccination Needs Index to Prioritise Municipal Interventions in Brazil

**DOI:** 10.1111/tmi.70059

**Published:** 2025-11-13

**Authors:** Fredi Alexander Diaz‐Quijano, Isaac Negretto Schrarstzhaupt, Francieli Fontana Sutile Tardetti Fantinato, Lely Stella Guzmán‐Barrera, Julio Croda

**Affiliations:** ^1^ School of Public Health, Department of Epidemiology, Laboratório de Inferência Causal em Epidemiologia [Laboratory of Causal Inference in Epidemiology] – LINCE‐USP University of São Paulo São Paulo São Paulo Brazil; ^2^ Pan American Health Organization (OPAS/OMS) Brasília Federal District Brazil; ^3^ School of Public Health, Post‐Graduate Program in Epidemiology University of São Paulo São Paulo São Paulo Brazil; ^4^ Immunization by the Pan American Health Organization Brasília Federal District Brazil; ^5^ Coordination of Immunization of Pan American Health Organization of PWR Brasília Federal District Brazil; ^6^ Faculdade de Medicina Universidade Federal de Mato Grosso do Sul Campo Grande Brazil

**Keywords:** immunisation prioritisation, public health policy, resource allocation, vaccination needs index (VNI), vaccine coverage

## Abstract

Addressing population health needs requires integrating indicators expressed on non‐comparable scales, particularly for vaccination, a cornerstone of public health.

**Objective:**

To propose and implement a Vaccination Needs Index based on national administrative data on tracer vaccines for children to support the prioritisation of Brazilian municipalities.

**Methods:**

In this ecological study, we developed and applied a Vaccination Needs Index using national administrative data from 5570 Brazilian municipalities (2018–2022), integrating transformed and standardised indicators of vaccination coverage and the number of susceptible children for three tracer vaccines: diphtheria, tetanus, and polio (DTP) (third dose), polio (third dose), and measles, mumps, and rubella (MMR) (second dose).

**Results:**

The estimated national coverages achieved in 2022 for the analysed vaccine doses were 79% for DTP, 67.7% for polio, and 46.1% for MMR. Consequently, approximately 3.0, 4.8 and 7.9 million children remained unvaccinated for these vaccines, respectively. When compared with criteria prioritising either unvaccinated children or low coverage alone, the top 5% of municipalities (*n* = 278) with the highest Vaccination Needs Index scores included a balanced mix of large, medium and small populations, with greater representation of the Legal Amazon region and Special Sanitary Indigenous Districts. These municipalities also had higher income inequality than the others, showing a mean difference in the Gini coefficient of 4.9 percentage points (95% CI: 4.2–5.7).

**Conclusions:**

The Vaccination Needs Index effectively integrated absolute and relative vaccination indicators, identifying populations with intersecting vulnerabilities and socioeconomic inequities. It provides a practical and scalable tool to guide tailored interventions and optimise resource allocation, thereby preventing the resurgence of vaccine‐preventable diseases.

**Abbreviations**
DSEI: Special Sanitary Indigenous Districts (Distritos Sanitários Especiais Indígenas)
DTP: Diphtheria‐Tetanus‐Pertussis
IBGE: Brazilian Institute of Geography and Statistics (Instituto Brasileiro de Geografia e Estatística)
MMR: Measles‐Mumps‐Rubella
PAHO: Pan American Health Organization
VNI: Vaccination Needs Index
WHO: World Health Organization

## 1 Introduction

Acknowledging health needs often requires the integration of several indicators that are expressed in non‐comparable scales [1, 2]. Some indexes have been used to represent health needs by aggregating standardised variables, usually after they are transformed to obtain distributions that, when compared, depict the variation of the population according to domains of interest [2]. The resulting metrics can then be used to prioritise funding and resource allocation for integral health approaches or to reorient intervention strategies for the population's access to services [3].

Concerning vaccination needs, prioritisation should take into account the coverage of the vaccines identified by PAHO‐WHO as tracers for children under 1 year of age and one‐year‐olds according to the basic schedule worldwide. In addition to considering several vaccines, another challenge is the nature of the indicator. Indicators such as the number of unvaccinated children (susceptible) or the vaccination coverage could be considered.

The absolute number of unvaccinated people directly represents the resource needs and affects the national indicators. However, the prioritisation based on an absolute indicator would end up focusing on large capitals and systematically excluding smaller cities even if they have lower vaccine coverage [4]. On the other hand, the prioritisation based exclusively on vaccination coverage would not consider the importance of the number of unvaccinated children and would undermine large groups of them that exist in big cities [5].

In this study, we proposed and applied a method to obtain an index of vaccination needs (VNI) considering three tracer vaccines of the routine schedule expected to be administered in the first 2 years of life, integrating the absolute number of unvaccinated children and the corresponding vaccination coverage. For this, we considered the complete primary schedule (3 doses) for Polio and Diphtheria‐Tetanus‐Pertussis (DTP) and two doses for Measles‐Mumps‐Rubella (MMR) vaccine in children born between 2018 and 2022, using open data from Brazil's National Immunisation Program.

## 2 Materials and Methods

In this ecological study, we proposed the construction of an index based on data recorded in the country's official vaccination information system, following steps similar to those that have been used to construct other social science indexes [6, 7]. The steps include the selection of the component indicators, data gathering, and the evaluation of the distributions, transformation, standardisation, aggregation, and application to establish an order of the analysis units.

### 2.1 Indicator Selection

As available and conceptually relevant for public health, we considered tracers the primary schema, including three doses for DTP and Polio vaccines and two doses for MMR in children up to 4 years of age in Brazil for the year 2022. Thus, we chose the following indicators:

- The number of unvaccinated children: In each city, we calculated the apparent number of children by age without the doses considered in the primary scheme.
- Vaccine coverage: In each city, we also calculated the proportion of children with the primary schema doses of each vaccine.

### 2.2 Data Source and Indicator Calculation

To calculate the vaccination coverage, the numerator and denominator were, respectively, the administered doses and the population for each age. Administered doses were obtained from the TABNET database, which is a platform with open health data. Filters to select the immunobiological corresponding to each vaccine were defined in consensus with the National Immunisation Program (Box S1). Population estimates were obtained from the National Institute of Geography and Statistics (IBGE) and the Brazilian Ministry of Health.

For each reference dose of the vaccines $\left(v\right)$, the administered doses expected to accumulate in each cohort defined by simple ages in each municipality were added. Each age‐specific cohort included the doses administered to that same birth cohort in previous years. For example, by the end of 2022, the three‐year‐old cohort should consider the doses administered to children aged three in 2022, two in 2021, one in 2020, and zero in 2019, and so on. The ratio between the sum of those doses and the population estimates, for each age and municipality ($P_{i,m}$: population of the age group “*i*” in the municipality “*m*”), was considered the specific coverage for each age‐municipality $\left(C_{v,i,m}\right)$ and was truncated to 100%. The number of unvaccinated (susceptible) children specific to each year of age and municipality ($S_{v,i,m}$) was calculated as $S_{v,i,m}=\left(1-C_{v,i,m}\right)\times P_{i,m}$.

The coverage in the 0–4‐year‐old group $\left(C_{v,0-4,m}\right)$ was calculated as the complement of the proportion of unvaccinated children in the corresponding age group. That proportion was calculated as the ratio between the sum of unvaccinated children for each age and the sum of the resident population for the same age. For example, the DTP vaccination coverage in the population from 0 to 4 years of age in each municipality was calculated as:

$$C_{\mathrm{DTP},0-4,m}=1-\frac{\sum_{i=0}^{i=4}S_{\mathrm{DTP},i,m}}{\sum_{i=0}^{i=4}P_{i,m}}.$$

The absolute indicator would be the sum of unvaccinated children ($S_{m,0-4,\mathrm{DTP}}$), which would correspond to:

$$S_{\mathrm{DTP},0-4,m}=\sum_{i=0}^{i=4}S_{\mathrm{DTP},i,m}.$$

### 2.3 Vaccination Needs Index (VNI) Construction

The indicators of each vaccine were graphically analysed to evaluate its distribution in the 5570 Brazilian municipalities. Both the number of unvaccinated children and the vaccination coverage had asymmetrical distributions, for which transformations were explored to minimise the difference between those distributions and a normal shape. The number of unvaccinated children was transformed by adding a single unit and obtaining the natural logarithm of that result:

$$\ln\left(S_{v,0-4,m}+1\right).$$

For the vaccination coverages, beyond the search for symmetry, the transformation included the inversion of the signal so that the sense could be the same as the indicator of susceptible children. The chosen transformation was:

$$\ln\left(\left(C_{v,0-4,m}\times -1\right)+2\right)=\ln\left(2-C_{v,0-4,m}\right).$$

Each resulting variable was standardised by subtracting the average and dividing by its standard deviation. In this way, we obtained transformed and standardised measures (or *Z*‐scores) for each absolute and relative indicator of each vaccine, all with a mean equal to zero and a standard deviation equal to one. As we summed the indicators of each vaccine, we obtained specific VNIs. Thus, for each vaccine (*v*), the vaccine‐specific VNI was calculated as:

$${\mathrm{VNI}}_{v}=Z_{\ln\left(S_{v,i,m}+1\right)}+Z_{\ln\left(2-C_{v,0-4,m}\right),}$$

where *Z* denotes the *Z*‐score of the transformed variable.

A consolidated VNI was then obtained as the sum of the vaccine‐specific VNIs for DTP, polio, and MMR:

$$\text{Consolidated}\;\mathrm{VNI}={\mathrm{VNI}}_{\mathrm{DTP}}+{\mathrm{VNI}}_{\text{Polio}}+{\mathrm{VNI}}_{\mathrm{MMR}}.$$

In summary, the construction of the VNI includes logarithmic transformations to reduce the disproportionate dominance of a small number of municipalities with extreme values, and a standardisation step to ensure that both the number of unvaccinated children and vaccination coverage contribute equally to the final index.

### 2.4 Validation

In the absence of a gold standard to validate the VNI, we focused on assessing characteristics that we considered to be expected in a group of prioritised municipalities, such as diversity in population size or challenging situations that hinder achieving high coverage. Thus, we described the characteristics of the municipalities that would be prioritised for belonging to the 5% (*n* = 278) with the highest VNI. As alternative references, we also described the characteristics of municipalities chosen with two other criteria of prioritisation:

1. The 278 cities with the highest susceptible (unvaccinated) average, considering the three vaccines studied.
2. The 278 cities with the lowest coverage average of the same vaccines.

Regarding the characteristics assessed, we expected that the prioritised group included both large and small cities, with a high number of unvaccinated children or low vaccination coverages. The size of municipalities was classified according to population estimates as “large” (> 100,000 inhabitants), “medium” (between 25,000 and 100,000 inhabitants), or “small” (< 25,000 inhabitants). Additionally, we expected that populations with more challenges to obtain high vaccination coverage, such as border municipalities, Legal Amazon cities, and Special Indigenous Sanitary Districts (DSEIs: Distrito Sanitário Especial Indígena) [8, 9], have a good chance of being prioritised. The Legal Amazon is a territory defined by Law 1806 of 01/06/1953, which includes 772 municipalities in nine Brazilian states [10]. The DSEI is the decentralised structure that answers to the management of the Indigenous Health System in Brazil. There are currently 34 DSEIs defined according to the geographical occupation of indigenous communities, which do not follow the political State division [11]. Finally, as the cities with more vaccination needs may also be the ones with greater socioeconomic inequality [12, 13], we compared the Gini coefficient between prioritised and non‐prioritised municipalities. The Gini coefficient measures inequality in income distribution on a scale from zero to one [14, 15]. A value of zero reflects perfect equality, where all income or assets are equal, while a Gini coefficient of one (or 100%) reflects the greatest inequality.

After, as an example of application, we illustrated how cutoff points could be used to categorize municipalities according to their VNI scores. Specifically, we suggest classifying the 200 municipalities with the highest VNI values as high priority, the next 1000 as moderate priority, and the remaining ones as standard priority. We deliberately avoided the term ‘low priority’, as maintaining appropriate vaccination levels requires continued routine efforts, and these municipalities should not be neglected.

### 2.5 Sensitivity Analysis

To assess the robustness of the VNI's characteristics, we conducted two sensitivity analyses:

1. When vaccination data for 2023 became available, we recalculated the VNI and assessed its concordance with that calculated for 2022 using the Spearman correlation coefficient. This was intended to assess the stability of the prioritisation criteria in the medium term.
2. We also calculated an alternative VNI by substituting the DTP and polio vaccines with the first dose of the yellow fever vaccine and the second dose of the pneumococcal vaccine. We then evaluated whether selecting the top 5% of municipalities according to this index would still better identify municipalities with a balanced distribution of size and other relevant characteristics than using absolute or relative criteria alone.

### 2.6 Research Ethics Approval: Human Participants

This study utilised open‐access data available in Brazil, which, according to Resolution 510/2016, did not require approval from an ethics committee, as the research did not involve direct interaction with human participants or collecting identifiable private information.

## 3 Results

For the year 2022, Brazil had approximately 14.7 million children between 0 and 4 years old (more than 2.9 million children in each single age category). In this population, we estimated coverage rates for the DTP (three doses), polio (three doses), and MMR (two doses) vaccines to be 79%, 67.7%, and 46.1%, respectively. Consequently, we calculated the number of unvaccinated children of approximately 3 million, 4.8 million, and 7.9 million for these vaccines, respectively. Although the number of unvaccinated children had medians that ranged from 132 to 490, this indicator reached into the hundreds of thousands (Table 1). Specifically, in São Paulo (SP) city, we calculated 149,754 children without the third dose of DTP, 268,849 without the third dose of polio, and 322,336 without the second dose of MMR.

**TABLE 1:** Vaccination coverage and unvaccinated children estimated for Diphtheria‐Tetanus‐Pertussis, Polio and Measles‐Mumps‐Rubella vaccines, Brazil, 2022.

Indicator	National values	Distribution measures on 5570 cities	
		Median (interquartile range)	Min; Max
0‐4 years old	14,703,269	854 (365–1929)	37; 779,817
Coverage			
DTP (3rd dose)	79.0%	83.1% (71.8%–92.3%)	20.8%; 100%
Polio (3rd dose)	67.7%	70.5% (63.6%–76.4%)	18.9%; 100%
MMR (2nd dose)	46.1%	40.4% (25.3%–55.7%)	0; 83.3%
Unvaccinated children			
DTP (3rd dose)	3,086,724	132 (36–404)	0; 149,754
Polio (3rd dose)	4,755,128	256 (105–626)	0; 268,849
MMR (2nd dose)	7,931,270	490 (219–1154)	13; 322,336

Abbreviations: DPT: Diphtheria‐Tetanus‐Pertussis; MMR: Measles‐Mumps‐Rubella.

**FIGURE 1:**
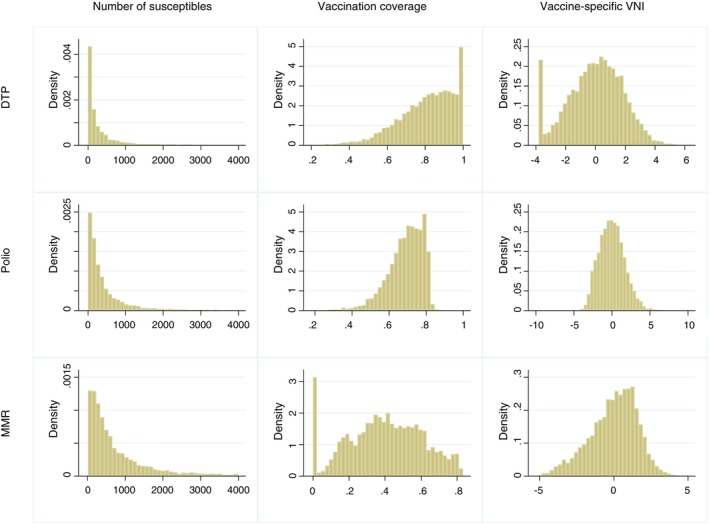
Distribution of number of unvaccinated children, vaccination coverage, and specific Vaccination Needs Index for Diphtheria‐Tetanus‐Pertussis, Polio, and Measles‐Mumps‐Rubella in Brazil, 2022.

Both the distribution of unvaccinated children and the coverage of these vaccines were highly asymmetric across Brazilian municipalities (left and centre columns of Figure 1). However, with the transformations, we obtained more symmetric vaccine‐specific VNIs (Figure 1, right column). The absolute indicators of the different vaccines were strongly correlated with each other, but the correlation between absolute and relative indicators was weaker (Table S1).

**FIGURE 2:**
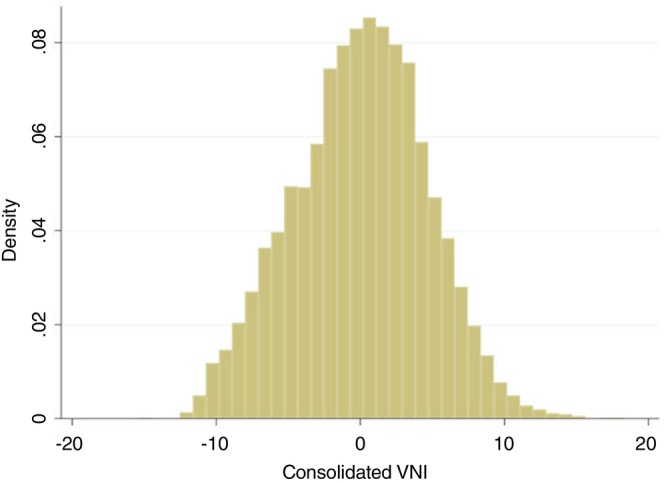
Distribution of a consolidated Vaccination Needs Index calculated for 5570 municipalities in Brazil, 2022.

When consolidated into a single index, we observed an even more symmetric distribution, resembling a normal distribution (Figure 2). The consolidated VNI values ranged from −15.3 to +18.4, and a cutoff of > 7.425 identified the top 5% (*n* = 278) of municipalities with the highest VNI values.

When comparing prioritisation strategies, the 278 municipalities corresponding to the top 5% with the highest average number of unvaccinated children were primarily large and did not include small ones. On the other hand, the 278 municipalities with the lowest coverage were mainly small (76.3%). Conversely, the group with the highest VNI had more similar proportions of large, medium, and small municipalities. Additionally, the group with the highest VNI had a higher frequency of municipalities located in the Legal Amazon region and Special Sanitary Indigenous Districts. The number of border cities was similar between the groups chosen based on coverage and VNI (21 and 20, respectively) but higher than the group selected based on the number of unvaccinated children (Table 2).

**TABLE 2:** Validation of methods to prioritise populations according to vaccine needs.

Characteristics of the municipalities	All municipalities (*n* = 5570)	Criterion for selection of 5% of municipalities (*n* = 278)		
		Highest susceptible average	Lowest vaccine coverage	Highest VNI
Size^a^				
Large	326 (5.9%)	249 (89.6%)	13 (4.7%)	109 (39.2%)
Medium	1118 (20.1%)	29 (10.4%)	53 (19.1%)	103 (37.1%)
Small	4126 (74.1%)	0 (0%)	212 (76.3%)	66 (23.7%)
Legal Amazon	772 (13.9%)	53 (19.1%)	79 (28.4%)	90 (32.4%)
Special sanitary indigenous districts	481 (8.6%)	48 (17.3%)	41 (14.7%)	58 (20.9%)
Border city	588 (10.6%)	13 (4.7%)	21 (7.6%)	20 (7.2%)
Sum of unvaccinated (susceptible) children				
Without 3rd dose for DTP	3,086,724	1,664,860	375,698	1,450,212
Without 3rd dose for polio	4,755,128	2,529,377	380,881	1,906,184
Without 2nd dose for MMR	7,931,270	3,856,877	526,644	2,723,163
Gini coefficient (%)–median (IQR)	50.3 (45.9–54.6)	53.6 (49–57.7)	52.9 (48.1–57.6)	54.9 (50–59.2)
Mean difference of the Gini coefficient (95% CI)		3.4 (2.6–4.2)	2.9 (2.1–3.7)	4.9 (4.2–5.7)

Abbreviations: DPT: Diphtheria‐Tetanus‐Pertussis; MMR: Measles‐Mumps‐Rubella; VNI: Vaccination Needs Index.

^a^ Municipalities were classified according to population estimates as large (> 100,000 inhabitants), medium (between 25,000 and 100,000 inhabitants) or small (< 25,000 inhabitants).

The number of children without the reference doses was higher in municipalities chosen based on the number of unvaccinated children (as expected). However, municipalities with the highest VNI had relatively close values to this latter group. On the other hand, municipalities prioritised due to their low coverage had a much smaller number of unvaccinated children. Moreover, regarding inequality, municipalities chosen based on VNI had a higher average Gini coefficient, which differed by 4.9% from the rest of the municipalities. Although the other two prioritisation criteria also led to differences in the Gini coefficient, these differences were smaller than those observed with the VNI criterion (Table 2).

**FIGURE 3:**
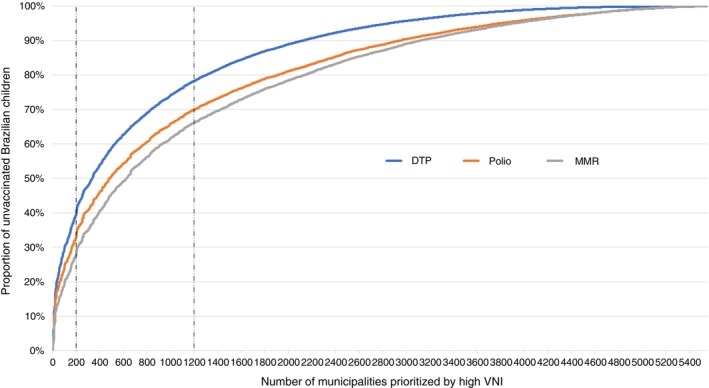
Proportion of children without reference doses by number of municipalities prioritised using the VNI.

In the prioritisation example using three categories, we again observed a better balance in municipality size within the high‐ and moderate‐priority categories compared with the standard‐priority category (Table S2). Additionally, these two categories included a higher proportion of municipalities located in the Legal Amazon region and those with Special Indigenous Health Districts than the standard‐priority category. Interestingly, the high‐ and moderate‐priority categories together, comprising 1200 of the 5570 municipalities, accounted for more than 65% of unvaccinated children for each vaccine analysed nationwide (Figure 3).

When the VNI calculated up to 2022 was compared with that up to 2023, the correlation was strong (Spearman coefficient = 0.71). On the other hand, when we calculated the alternative VNI integrating indicators for MMR, yellow fever, and pneumococcal vaccines, we observed comparable results, including a symmetrical index and a more balanced distribution of municipality sizes compared with other criteria (Figure S1 and Table S3). Additionally, the top 5% of municipalities with the highest alternative VNI included a greater proportion of populations from the Legal Amazon region and Special Indigenous Health Districts, as well as a larger difference in the Gini coefficient compared with other prioritisation criteria.

## 4 Discussion

Prioritisation is essential for the efficient use of public health resources. However, choosing a metric to prioritise populations by vaccination needs is a methodological challenge. Absolute indicators, such as the number of unvaccinated individuals, are directly related to the resource requirements (including the number of doses and healthcare personnel). On the other hand, the relative measure of coverage is important to avoid the exclusion of remote communities. Additionally, to achieve herd or collective immunity, the number of immune individuals should be distributed evenly [16, 17], so achieving and maintaining high coverage in all municipalities can prevent the formation of susceptible clusters where outbreaks can begin. Moreover, in smaller municipalities with low coverage, interventions with fewer resources can have a relatively greater impact by reducing the risk of vaccine‐preventable diseases.

The proposed VNI integrated absolute and relative indicators of essential vaccines. In the validation, this index allowed for greater representation within the prioritised group of municipalities with characteristics that suggest vulnerability, such as those with indigenous districts or regions in border areas, which also encompass a high number of unvaccinated people. In addition, the VNI outperformed other evaluated prioritisation strategies in identifying a group of municipalities that differed from the rest in terms of the Gini coefficient.

The VNI was calculated using coverage rates based on cumulative doses expected for each age‐specific cohort, accounting for doses administered in previous years. This cohort‐based approach differs from the conventional method, which sums doses marginally, and explains some discrepancies between the vaccination coverage estimates in this study (DTP: 79.0%, Polio: 67.7%, MMR: 46.1%) and those reported by the WHO for Brazil in 2022 (DTP: 77.16%, Polio: 74.94%, Measles‐containing vaccine: 57.57%) [18], which are based solely on the target population for each calendar year. Our approach provides a more accurate reconstruction of vaccination indicators by considering both population estimates and doses administered across multiple years.

Regarding its application, the VNI can be used in a variety of ways. Thus, several categories can be established for intervention levels. As we see in our example, the 200 municipalities with the highest VNI covered approximately one‐third of the unvaccinated population with the reference doses (Figure 3). On the other hand, by adding the following 1000 cities, a total of 1200 priority municipalities with the highest VNI accounted for approximately two‐thirds of the population without the reference doses for DTP, polio, and MMR in the country. In this way, decision‐makers can apply different categorizations to plan activities with varying levels of intervention and efficiently enhance vaccination access for the target population.

For example, municipalities classified in the highest priority tier warrant intensive, tailored outreach. This could include deploying mobile vaccination units, implementing active house‐to‐house tracking of unvaccinated children, and collaborating with community leaders to identify and overcome specific access barriers (e.g., geographical, informational, or cultural). For a moderate priority tier, the focus may shift to strengthening routine activities, such as extending health post hours, conducting strategic ‘catch‐up’ campaigns in vulnerable neighbourhoods, and enhancing community mobilisation. Finally, municipalities in the standard priority tier should be encouraged to maintain their routine immunisation services vigilantly, ensuring continuous vaccine supply and monitoring coverage to prevent declines. This tiered approach ensures that finite resources are directed where the need and potential impact are greatest, while sustaining the overall performance of the immunisation program.

When applying the VNI with updated information, we observed that the correlation between two consecutive years was strong [19]. We believe it is necessary to periodically update the estimates, as the vaccination situation can change yearly due to various factors, including public health actions and population behaviours. However, we interpreted the strong correlation as suggesting that the order may be stable, allowing VNI‐based prioritisation to plan interventions in the medium and long term.

On the other hand, when we changed the vaccines to include yellow fever and pneumococcus, the VNI maintained desirable traits, such as prioritising municipalities with a balanced size and features that suggest difficulties for vaccination. For all the above reasons, we interpreted the sensitivity analysis as indicating that the VNI is a robust tool for prioritisation.

We consider that the limitations of the proposed VNI are linked to those of the indicators used for its construction, as well as the data recorded in the national vaccination information system. In particular, the number of doses may not necessarily represent the number of immunised individuals. Without an individualised registry of people, corrections cannot be performed (like those for mortality) for vaccinated and unvaccinated individuals. However, we expect the classification errors to be non‐differential and thus not affect the final prioritisation order.

In this study, the VNI was based on vaccines from the basic immunisation schedule for children, whose absolute indicators were strongly correlated. We considered this high correlation expected, as the index is intended to reflect conditions influenced by common determinants. Nevertheless, the VNI could also be constructed using less correlated vaccine indicators to enhance complementarity. Interestingly, although the absolute indicators of different vaccines were highly correlated, their correlation with the corresponding relative indicators was much lower. This illustrates, from a statistical perspective, how combining absolute and relative indicators contributes to a more complementary and informative index.

The VNI is specifically designed for municipal‐level vaccination assessment and can be easily constructed using routinely collected data. Its novelty lies in combining two critical and readily available metrics, coverage (a relative measure) and the number of susceptible children (an absolute burden measure), into a single, actionable index. This makes the VNI less abstract than previously proposed social and equity models [20, 21, 22, 23], which address vaccination indirectly by focusing on its determinants.

Moreover, the VNI is more directly applicable to routine immunisation program management than complex supply‐chain optimizations [24, 25]. While other indexes composed of diverse indicators have been used in different scenarios, prioritisation for improving vaccination coverage has typically considered relative or absolute measures in isolation [20, 26, 27]. Existing mathematical models for vaccine prioritisation do not produce a single integrated index, highlighting the VNI's ability to synthesise the needs of the population.

The methodology underlying the VNI is flexible and can be extended to other populations or vaccines, depending on prioritisation requirements. By combining multiple vaccines, it addresses the needs of individuals rather than focusing on a single vaccine target. Furthermore, the methodology can be adapted to include additional populations or vaccines as needed in the prioritisation process. Although designed for sub‐national prioritisation within a country, the VNI framework can also be applied by other nations or to large regions within a country. However, direct cross‐country comparisons may be limited, mainly due to differences in the distributional parameters (i.e., means and standard deviations) used for standardizations.

Future research could explore the integration of the VNI with other models, for example, by using it to define priority areas for supply‐chain optimization or by combining it with a broader set of socioeconomic and health care indicators related to the determinants of low coverage [28]. In this way, the VNI can complement existing approaches and guide actions based not only on vaccination outcomes but also on the underlying factors that influence coverage.

In conclusion, the proposed VNI integrated indicators of different natures that represent the vaccination situation according to the scheme indicated by age. This approach can facilitate the identification of populations that need differentiated interventions to improve their health situation and prevent the resurgence of eliminated or controlled diseases that are preventable with vaccines. This method can be adapted and adopted by any country that, based on its databases and system, wants to prioritise its target populations at the municipal management level.

## Ethics Statement

The authors have nothing to report.

## Conflicts of Interest

The authors declare no conflicts of interest.

## Supporting information


**Box S1.** Selections in the applied doses website to build the initial database.
**Table S1:** Matrix of Pearson correlation coefficients between transformed absolute and relative indicators.
**Table S2:** Example of using the INV to categorise the priority level of Brazilian municipalities according to their vaccination needs (*n* = 5570).
**Figure S1:** Distribution of an alternative Vaccination Needs Index calculated for 5570 municipalities in Brazil, integrating indicators of MMR, Yellow Fever, and Pneumococcal vaccines, 2022.
**Table S3:** Validation of the alternative version of the VNI in comparison with other methods to prioritise populations.
