# Supplementary material for: Proposal of a Vaccination Needs Index to Prioritise Municipal Interventions in Brazil
Source: Trop Med Int Health. 2025 Nov 13;31(2):198–206. doi: 10.1111/tmi.70059 (PMC12868402; doi:10.1111/tmi.70059)
Supplement: Supplementary file 1 — Box S1. Selections in the applied doses website to build the initial database. Table S1: Matrix of Pearson correlation coefficients between transformed absolute and relative indicators. Table S2: Example of using the INV to categorise the priority level of Brazilian municipalities according to their vaccination needs (n = 5570). Figure S1: Distribution of an alternative Vaccination Needs Index calculated for 5570 municipalities in Brazil, integrating indicators of MMR, Yellow Fever, and Pneumococcal vaccines, 2022. Table S3: Validation of the alternative version of the VNI in comparison with other methods to prioritise populations. [file TMI-31-198-s001.docx]

**SUPPLEMENTARY MATERIAL**

| **Box S1. Selections in the applied doses website to build the initial database.** |
| --- |
| For all vaccines:   - Source: TABNET (<http://tabnet.datasus.gov.br/cgi/dhdat.exe?bd_pni/dpnibr.def> and <http://tabnet.datasus.gov.br/cgi/deftohtm.exe?ibge/cnv/popsvsbr.def>)   - Year: 2008 to 2022   - We selected “municipality” as the observations and “age rate” as the variables (columns) |
| - Specific for Polio:   - Immunobiological:     - Inactivated Poliovirus Vaccine (IPV)     - Oral Poliovirus Vaccine (OPV)     - OPV/IPV (sequential)     - Inactivated Pentavalent (DTPa/Hib/IPV)     - Hexavalent (DTPa/Hib/HB/IPV)   - Doses: 1, 2, 3, 1^st^ booster and 2^nd^ booster. |
| - Specific for DTP:   - Immunobiological:     - Hexavalent (DTPa/Hib/HB/IPV)     - Pentavalent (DTP/Hib/HB)     - Tetravalent (DTP/Hib)     - Diphtheria-Tetanus-Acellular Pertussis (DTPa)     - DTP     - Inactivated Pentavalent (DTPa/Hib/VIP)     - DTPa   - Doses: 1, 2, 3, 1st booster and 2nd booster |
| - Specific for MMR:   - Immunobiological:     - MMR (SCR);     - Tetra viral (Measles, Mumps, Rubella, and Varicella);   - Doses:     - Dose 1: Dose 1 of MMR and Dose 1 of Tetraviral     - Dose 2: Dose 2 of MMR, Single dose of Tetraviral and Dose 2 of Tetraviral |
| Estimated population data from the Health Ministry were obtained in:   - Source: TABNET (<http://tabnet.datasus.gov.br/cgi/deftohtm.exe?ibge/cnv/popsvsbr.def>)   Year: 2021 (latest estimative that were available at the time of the study) |

| **Table S1. Matrix of Pearson correlation coefficients between transformed absolute and relative indicators.** | | | | | | | |
| --- | --- | --- | --- | --- | --- | --- | --- |
| Vaccine indicators | | Absolute | | | Relative | | |
|  |  | DTP | Polio | MMR | DTP | Polio | MMR |
| Absolute | DTP | 1.00 |  |  |  |  |  |
|  | Polio | 0.84 | 1.00 |  |  |  |  |
|  | MMR | 0.80 | 0.96 | 1.00 |  |  |  |
| Relative | DTP | 0.69 | 0.41 | 0.35 | 1.00 |  |  |
|  | Polio | 0.62 | 0.38 | 0.30 | 0.95 | 1.00 |  |
|  | MMR | 0.27 | 0.05 | 0.24 | 0.47 | 0.46 | 1.00 |

| **Table S2: Example of using the INV to categorize the priority level of Brazilian municipalities according to their vaccination needs (n=5570).** | | | | |
| --- | --- | --- | --- | --- |
| **Characteristics of the municipalities** | **All municipalities (n=5,570)** | **Priority category according to the VNI*** | | |
|  |  | **High**  **(n=200)** | **Moderate**  **(n=1000)** | **Standard**  **(n=4370)** |
| Size† |  |  |  |  |
| Large | 326 (5.9%) | 45 (22.5%) | 463 (46.3%) | 3618 (82.8%) |
| Medium | 1118 (20.1%) | 75 (37.5%) | 373 (37.3%) | 670 (15.3%) |
| Small | 4126 (74.1%) | 80 (40%) | 164 (16.4%) | 82 (1.9%) |
| Legal Amazon | 772 (13.9%) | 75 (37.5%) | 219 (21.9%) | 478 (10.9%) |
| Special Sanitary Indigenous Districts | 481 (8.6%) | 44 (22%) | 136 (13.6%) | 301 (6.9%) |
| Border city | 588 (10.6%) | 13 (6.5%) | 72 (7.2%) | 503 (11.5%) |
| Sum of unvaccinated (susceptible) children |  |  |  |  |
| Without 3^rd^ dose for DTP | 3,086,724 | 1,217,358 | 1,198,328 | 671,038 |
| Without 3^rd^ dose for polio | 4,755,128 | 1,551,737 | 1,772,225 | 1,431,166 |
| Without 2^nd^ dose for MMR | 7,931,270 | 2,195,567 | 3,048,415 | 2,687,288 |
| Gini coefficient (%) – mean (95% CI) ‡ | 50.3 (50.1 – 50.5) | 55.2 (54.2 – 56.2) | 53 (52.6 – 53.4) | 49.5 (49.3 – 49.7) |

VNI: Vaccination Needs Index; DPT: Diphtheria-Tetanus-Pertussis; MMR: Measles-Mumps-Rubella; CI: Confidence Interval.

* In this example, we considered municipalities with VNI > 8.053 as high-priority, those with VNI between 3.651 and 8.053 as moderate-priority, and those with VNI < 3.651 as standard-priority.

† Municipalities were classified according to population estimates as large (>100,000 inhabitants), medium (between 25,000 and 100,000 inhabitants), or small (<25,000 inhabitants).

‡The mean Gini coefficients differed significantly between categories (p-values <0.0001 for all pairwise comparisons).

**Figure S1. Distribution of an alternative Vaccination Needs Index calculated for 5,570 municipalities in Brazil, integrating indicators of MMR, Yellow Fever, and Pneumococcal vaccines, 2022.**


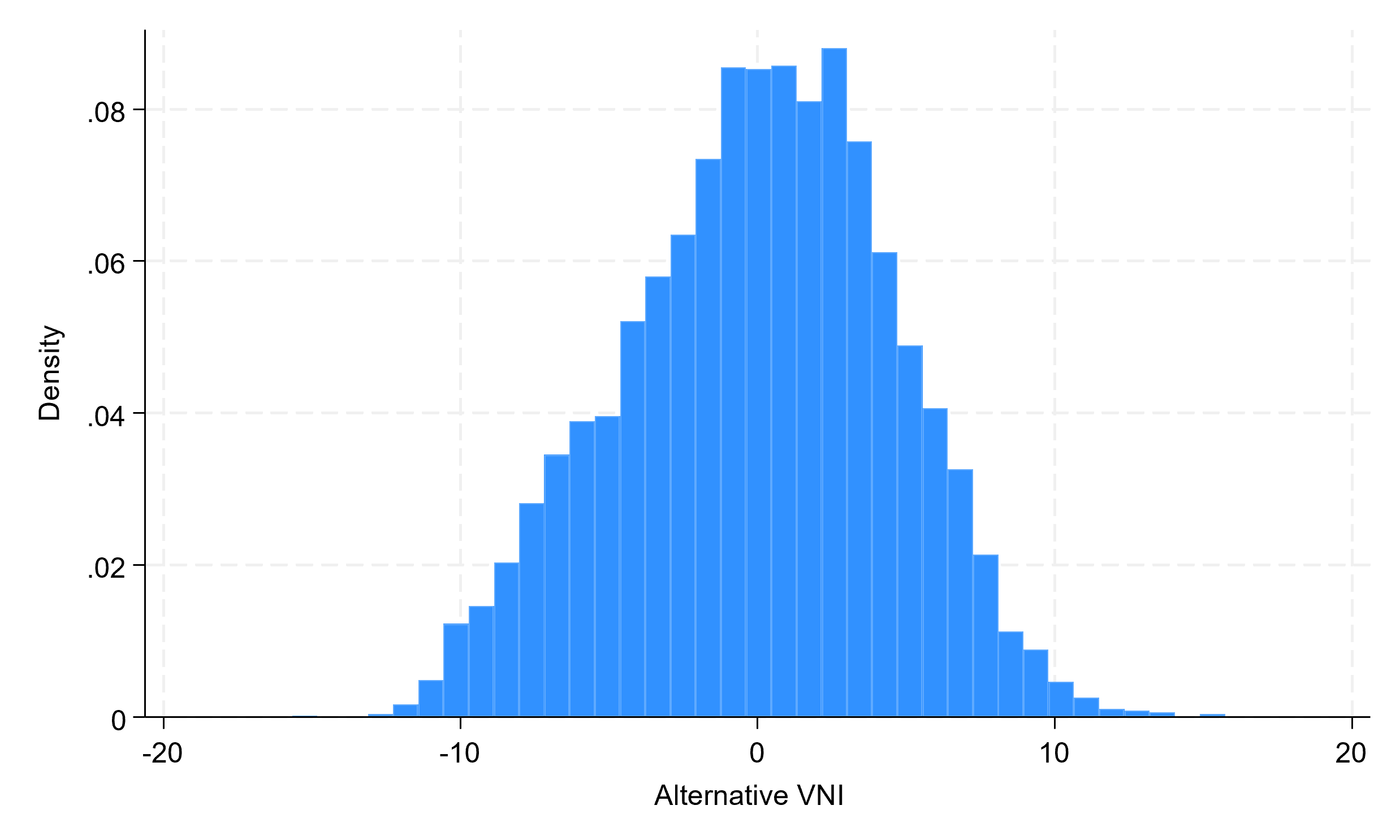


| **Table S3: Validation of the alternative version of the VNI in comparison with other methods to prioritize populations.** | | | | |
| --- | --- | --- | --- | --- |
| **Characteristics of the municipalities** | **All municipalities (n=5,570)** | **Criterion for selection of 5% of municipalities (n=278)** | | |
|  |  | **Highest susceptible average** | **Lowest vaccine coverage** | **Highest VNI** |
| Size* |  |  |  |  |
| Large | 326 (5.9%) | 253 (91%) | 11 (4%) | 131 (47.1%) |
| Medium | 1118 (20.1%) | 25 (9%) | 56 (20.1%) | 108 (38.8%) |
| Small | 4126 (74.1%) | 0 (0%) | 211 (75.9%) | 39 (14%) |
| Legal Amazon | 772 (13.9%) | 50 (18%) | 84 (30.2%) | 94 (33.8%) |
| Special Sanitary Indigenous Districts | 481 (8.6%) | 46 (16.5%) | 39 (14%) | 60 (21.6%) |
| Border city | 588 (10.6%) | 13 (4.7%) | 23 (8.3%) | 22 (7.9%) |
| Sum of unvaccinated (susceptible) children |  |  |  |  |
| Without 2^nd^ dose for MMR | 7,931,270 | 3,857,707 | 511,561 | 2,978,380 |
| Without 1^st^ dose for YF | 8,932,469 | 4,830,872 | 530,324 | 3,758,278 |
| Without 2^nd^ dose of the Pneumococcal | 2,939,071 | 1,533,974 | 347,289 | 1,438,590 |
| Gini coefficient (%)– median (IQR) | 50.3 (45.9 – 54.6) | 53.3 (49 – 57.5) | 53.1 (48.3 – 57.6) | 54.8 (49.8 – 59.5) |
| Mean difference of the Gini coefficient (95% CI) | | 3.2 (2.4 – 4) | 3.2 (2.4 – 4) | 5 (4.2 – 5.8) |

VNI: Vaccination Needs Index; MMR: Measles-Mumps-Rubella; YF: Yellow fever; CI: Confidence Interval.

* Municipalities were classified according to population estimates as large (>100,000 inhabitants), medium (between 25,000 and 100,000 inhabitants), or small (<25,000 inhabitants).
